# ‘Extreme’ organisms and the problem of generalization: interpreting the Krogh principle

**DOI:** 10.1007/s40656-018-0231-0

**Published:** 2018-10-31

**Authors:** Sara Green, Michael R. Dietrich, Sabina Leonelli, Rachel A. Ankeny

**Affiliations:** 1 Department of Science Education, University of Copenhagen, Copenhagen, Denmark; 2 Department of History and Philosophy of Science, University of Pittsburgh, Pittsburgh, USA; 3 Department of Sociology, Philosophy and Anthropology, University of Exeter, Exeter, UK; 4 School of Humanities, University of Adelaide, Adelaide, Australia

**Keywords:** August Krogh principle, Experimental organisms, Comparative physiology, Adaptation, Generalization, Model organisms

## Abstract

Many biologists appeal to the so-called Krogh principle when justifying their choice of experimental organisms. The principle states that “for a large number of problems there will be some animal of choice, or a few such animals, on which it can be most conveniently studied”. Despite its popularity, the principle is often critiqued for implying unwarranted generalizations from optimal models. We argue that the Krogh principle should be interpreted in relation to the historical and scientific contexts in which it has been developed and used. We interpret the Krogh Principle as a heuristic, i.e., as a recommendation to approach biological problems through organisms where a specific trait or physiological mechanism is expected to be most distinctively displayed or most experimentally accessible. We designate these organisms “Krogh organisms”. We clarify the differences between uses of model organisms and non-standard Krogh organisms. Among these is the use of Krogh organisms as “negative models” in biomedical research, where organisms are chosen for their *dissimilarity* to human physiology. Importantly, the representational scope of Krogh organisms and the generalizability of their characteristics are not fixed or assumed but explored through experimental studies. Research on Krogh organisms is steeped in the comparative method characteristic of zoology and comparative physiology, in which studies of biological variation produce insights into general physiological constraints. Accordingly, we conclude that the Krogh principle exemplifies the advantages of studying biological variation as a strategy to produce generalizable insights.

## Introduction

Classical physiologists, such as Claude Bernard (1813–1878) and August Krogh (1874–1949), emphasized the importance of organism choice for observational, experimental, and comparative studies in biological research. Krogh’s 1929 formulation of this idea has become known as the Krogh principle, which claims that: “for a large number of problems there will be some animal of choice, or a few such animals, on which it can be most conveniently studied” (Krogh 1929). The Krogh principle has been widely invoked to justify organism choice across all areas of biology, and yet its use is open to diverse interpretations. Some of these have led to reflections upon the limitations and possible biases of the principle.

On the one hand, the principle is sometimes taken to assert the importance of identifying ideal or optimal models for answering all questions about a given physiological problem (e.g., Feder and Watt 1992; Wayne and Staves 1996; Randall et al. 1997). On the other hand, the principle has sometimes been interpreted as validating the application of a claim produced in relation to one optimal or optimized model organism to other species. When it is thus taken as a justification for generalizing the scope of biological claims, an obvious limitation of the principle is the risk of warranting generalizations from the study of species that are not representative of the physiology or functional organization of other types of organisms (Holmes 1993). In other words, the organisms picked out using the Krogh principle may be “special cases” rather than ideal models. As a result, when the Krogh principle is taken as referring to the choice of organisms well-suited to investigating specific problems, a tension arises with regard to the goal of making generalization from those specific cases to other organisms (Gest 1995; Holmes 1993; Krebs and Krebs 1980).

Krebs and Krebs (1980) exemplify the problem of extrapolating from one species to another with reference to a controversy that took place in the 1950s and 1960s. At this time, biologists debated whether the dynamics of animal population size is dependent on the density of the population, with one group arguing strongly for density dependence and the other group rejecting this claim. Krebs and Krebs regard the debate as futile because it resulted from problematic extrapolations from studies of different animals (birds and insects) that respond to different physiological and environmental constraints. They therefore caution against the application of the Krogh principle to complex problems and recommend that the principle should only be used for analyses at the molecular level where the potential for generalization may be higher. The applicability of the Krogh principle has also been central to discussions about the use of animal experiments in biomedical research. Some authors frame the problem as “the dilemma of which animal model(s) most accurately represent(s) and reproduce(s) the human condition being investigated” (Arnoczky et al. 2009: 32). While this problem is common to all animal experiments, the use of non-standard organisms following the Krogh principle may be particularly problematic as in their view it may lead to “fallacious generalizations”. These examples highlight how understanding the Krogh principle raises important questions about the scientific usefulness and epistemic status of non-standard experimental organisms.

In this paper, we argue that the utility of the Krogh principle does not depend on the level or complexity of the system examined, nor on the success rate of generalizations across species. Instead, we highlight the importance of interpreting the principle in the context of Krogh’s work and his main point in the 1929 lecture, namely to stress the importance of the comparative method and to study physiological processes in all their various forms in different organisms. Krogh’s much-cited lecture was intended as a response to an ongoing debate on the generalizability of animal models to human physiology. At this time, experimental physiology was still in its early phases and human physiology was mainly done by physicians in hospitals (Lindstedt 2014). The latter was motivated by the view that insights relevant for medicine could only be based on humans or closely related species, with the consequence that research areas such as zoology were considered largely irrelevant to medicine. On the other end of the spectrum of views in the debate, general physiologists argued that physiological principles could be generalized from studies of much simpler organisms.

Krogh’s view can be interpreted as an alternative or middle ground between these positions. Krogh had a strong interest in zoology, as well as physiology and medicine. After finishing the medical preparatory examination at University of Copenhagen (1893), Krogh completed a magister conference (2 years education) in zoology (Schmidt-Nielsen 1984). The experimental skills developed as part of that program made him an attractive assistant to the physiologist Christian Bohr (father of the physicist Niels Bohr), who became Krogh’s mentor through his doctoral studies. Even before finishing his dissertation, Krogh gave two lectures on what he described as “a kind of program declaration, designed to show what I understand by Zoophysiology (comparative physiology)” (Schmidt-Nielsen 1995: 49). The lectures were given in March 1901, and this was the first time Krogh presented what is now known as the comparative method. In these lectures, he not only argued for the relevance of comparative physiology for medicine, but also for the independence of the discipline.^1^

We argue that focusing on the historical and scientific context in which the Krogh Principle was originally formulated, and particularly the goals that it was meant to foster, helps to pinpoint the conditions under which the Krogh Principle may prove fruitful in guiding research practices. Through this analysis, we offer an alternative to a view of biological research as involving either an emphasis on the differences among organisms, and thus on the study of biological diversity (a ‘splitters’ approach), or a search for generalizable mechanisms and patterns that hold across diverse species (a ‘lumpers’ approach) (see also Gest 1995).

We begin in Sect. 2 by clarifying what is special about what biologists sometimes refer to as ‘Krogh organisms’ through a comparison of their common features with those of model organisms. This analysis is based on previous philosophical studies of both types of organisms (Love 2010; Ankeny and Leonelli 2011) as well as an examination of features highlighted in papers that explicitly refer to the Krogh principle. Importantly, the features that we identify are not considered intrinsic to specific organisms or stable across research contexts. Rather, we propose that the Krogh principle functions as a rule of thumb or a heuristic that frames specific organisms as the best-suited for experimental studies of specific physiological characteristics or mechanisms.^2^

One strategy used by authors who invoke the Krogh principle is to look for highly adaptive traits. We clarify why a focus on adaptations can be experimentally convenient by presenting a series of examples illustrating how specific adaptations can make the identification of solutions to physiological problems easier. We then return to the problem of generalization by looking at examples that connect a focus on adaptations to translational power within contexts of knowledge application (most typically human medicine). Our choice of examples in this context are inspired by contemporary research within comparative physiology, where Krogh organisms are sometimes selected for their ability to deal with physiological problems that lead to disease development in humans (Alstrup and Wang 2016). We explore the role of Krogh organisms as “negative models” in translational biomedical research in Sect. 3. We present two examples with different translational outcomes and discuss the merits of the Krogh principle on this basis. We argue that the utility of the principle does not depend in any strong sense on the potential for generalizations. Rather, it depends on how researchers explore the applicability of the experimental organism that is being targeted for understanding other species.

Our examination of historical and contemporary examples within physiology shows that the use of the Krogh principle is often tied to the comparative method. Section 4 further clarifies how this interpretation is supported by the historical context in which the Krogh principle was originally formulated. The comparative method exploits a productive tension between general constraints on physiological structure–function relations and biological diversity. We then discuss the relevance of ‘extreme organisms’ for biomedical research (Sect. 5). We conclude by highlighting that the strategy of exploring variation over common physiological themes exemplifies how the study of diversity is compatible with a quest for insights into general physiological mechanisms.

## The Krogh principle as a heuristic and the value of studying extreme adaptations

The Krogh principle highlights that many problems in physiology are more conveniently studied through specific choices of experimental animals. What constitutes “convenience” or “good choices”, however, may depend on several factors. Convenience may refer to the well-cited practical advantages associated with organismal maintenance in laboratories, such as cost-effectiveness, small size, short generation time, and so on, or to ethical or legal issues (Bolker 2009). Moreover, experimental convenience in the context of the Krogh Principle is often connected to a justification of the relevance of studying distinctive or extreme adaptations.^3^ This focus raises an important philosophical question about the extent to which studies of “aberrant creatures and freaks of nature” (Adriaens and Herrel 2009: 1) are relevant for insights into general and human physiology. Moreover, the focus on atypical or extreme morphologies makes Krogh organisms dissimilar to other types of experimental organisms—and particularly to model organisms—in interesting ways that require further philosophical analysis.

Traditional model organisms as designated by the National Institutes of Health (such as *Drosophila melanogaster*, *Caenorhabiditis elegans* and *Arabidopsis thaliana*) have been characterized by a wide representational scope, that is a capacity to represent a large number of other species. This representational scope results not only from genetic conservation but also from their experimental tractability, the extent to which they have been standardized to fit specific investigative procedures and lines of inquiry, and the development of data infrastructures supporting cross-species inferences. Model organisms are also typically viewed as having an extensive representational target, in the sense of allowing the study of a large number of diverse biological phenomena, and indeed for the ways in which data collected on different aspects of the same organism can be brought together to understand that organism as an integrated whole (Ankeny and Leonelli 2011; Leonelli and Ankeny 2012, 2013).

By contrast, Krogh organisms are typically not standardized; little effort is put into building specialized infrastructures around them; and they are often chosen for specialized features that allow for investigation of specific biological problems, rather than representing and connecting different aspects of the organism. In other words, Krogh organisms are chosen from a range of possible experimental organisms because they are particularly well-suited for identifying and studying a specific mechanism or physiological problem. Love (2010) frames this as the difference between what he calls a ‘representational requirement’ and a ‘problem focus’ in the use of certain experimental organisms. The former is typically ascribed to model organisms while the latter is characteristic of the choice of Krogh organisms. The identified problem solution sometimes turns out to be a generalizable mechanism, but unlike model organisms the representational scope of Krogh organisms is open-ended: while model organisms are expected to represent a wide variety of species, Krogh organisms can vary widely from representing only themselves to standing for several other species.

A concrete example provided by Krogh himself is that of a tortoise that he and Christian Bohr found particularly suitable for studies of the physiology of respiratory mechanisms in vertebrates (Krogh 1929). Bohr had introduced the tortoise to study lung function because the structure of its trachea allowed for independent determination of gas exchange in the two lungs. Compared to other vertebrates, the branching of the trachea into the main bronchi for each lung is situated unusually high up in the neck of this tortoise, making this animal ideal for respiratory studies. These studies, together with experiments on frogs, were central to Marie and August Krogh’s demonstration that gas exchange occurred by diffusion alone (and not active transport), a discovery for which August Krogh was awarded the Nobel Prize in 1920 (Krogh 1910a, b).^4^ As this example illustrates, Krogh organisms are not selected for their representational scope or their similarity to other organisms; rather, they are selected because of distinct features that make a given trait (a mechanism in this case) more *experimentally accessible*. The unique features of the tortoise resulted in a discovery that then was generalizable to other species, despite the obvious differences in physiology. Importantly, however, generalizability of the findings was not a fundamental condition which, if refuted, would put the choice of organism in question or dramatically distort the credibility of the research done on it. We shall return to this issue in Sects. 3 and 4.

Very often the traits that are selected as prime characteristics of Krogh organisms are noteworthy, distinctive, or even extreme adaptations. Indeed, the Krogh principle is often invoked when justifying a choice of focusing on organisms with specific adaptive traits. Sometimes adaptations are highlighted primarily as an experimentally convenient access point to physiological mechanisms. In such contexts, the focus on adaptations is instrumental in the sense that it is tied to practical aspects of experimental convenience. For instance, Krogh’s tortoise with the unusual tracheal morphology exemplifies a distinctive adaptation that offered much easier experimental access to study respiratory mechanisms, as compared to other vertebrates. The adaptation is experimentally convenient because the structures are easier to isolate and manipulate. Another well-known example is the giant axon of the *Loligo* squid that allowed for voltage clamping experiments, making it possible to generate evidence for the Hodgkin-Huxley model of the action potential (Trumpler 1997; Schwiening 2012). A final example is Hans Krebs’s (1898–1945) work on the pathway of oxidative metabolism (the citric acid cycle), which was based on studies of pigeon breast muscles (Holmes 1991). Krebs (1975) explains this choice by highlighting the robustness of the mitochondria in these tissues. He views mitochondrial stability as an adaptation of the cells in the main flight muscle that makes these resilient not only to environmental stress but also to the experimental procedures of mincing and suspension. This feature, together with the increased metabolic activity, make this tissue well suited for studies of metabolic analysis. In all of these examples, adaptations provide experimental access to physiological mechanisms that would have been difficult, or maybe even impossible, to study by using other animals (Holmes 1993).

In other cases, the adaptive mechanism itself may be the focus of research, and organisms may be selected for study because of the adaptive trait that they display. In such cases, extreme adaptations provide clear examples of physiological structures and mechanisms that are capable of dealing with environmental challenges.^5^ A key idea is that adaptive traits studied among the ‘extremely adapted’ organisms can serve as a hypothesis for more general mechanisms or traits in other organisms that face similar, although less extreme, adaptive challenges. Species that are extremely well adapted to deal with specific environmental challenges are assumed to “show with special clarity the relationship between structure, function, and environment” (Lauder et al. 1995: 702).

As an illustration of this idea, consider studies of how desert rodents manage to survive in environments with sparse access to water which can give insights to relations between kidney morphology, function, and environmental needs. A structure called the loop of Henle is central to reabsorption of water in all mammalian kidneys. This structure can upregulate urea concentration through osmotic gradients along the loop. Deserts rodents, such as Australian hopping mice, have extraordinarily long loops of Henle which allow them to excrete very concentrated (hyperosmotic) urine (Schmidt-Nielsen 1983). Accordingly, studies of the morphology and physiological mechanisms found in organisms in extreme environments can give insights into “the limits to which organismal design can be driven and often best and most clearly illustrate the basic design principles at work” (Adriaens and Herrel 2009: 1). Moreover, an examination of how the structure varies among different organisms in different environments can give insights into the scope of physiological variation and adaptive demands (Logan 2002). It is therefore not surprising that physiology textbooks often discuss kidney structure and function through comparison of contrastive examples, for example through comparison of desert rats to aquatic freshwater mammals with exceptionally short loops of Henle (e.g., Campbell and Reece 2005: 939).

A central feature of the interest in extreme adaptations is that they are often conceptualized as offering easier access to the identification of physiological mechanisms and thus to understanding relations between structures, functions, and environmental demands. The heuristic associated with the Krogh principle can thus be reconstructed as follows: Krogh organisms serve as experimental access points for the identification and exploration of specific mechanisms or physiological problems. By selecting experimental animals where a trait is more accessible or prominent, causal relations between the trait and the environment are easier to pick out and hence make the identification of solutions to physiological problems clearer (see also Lindstedt 2014; Lindstedt and Nishikawa 2015). The analysis does not stop with extreme adaptions, however. A central part of the heuristic value of the Krogh principle involves *exploring variation* over the identified physiological features.

Interpreted as a heuristic, rather than an empirical claim, the principle does not assume that the identified traits or features are applicable or generalizable across species.^6^ Rather, the representational scope is itself an empirical question, which makes Krogh organisms different from standardized model organisms. But, as we shall discuss in Sect. 4, the open-endedness of the representational scope may become a problem for the Krogh principle whenever it is detached from the comparative method that Krogh originally emphasized. Before delving into the challenges posed by different applications of the knowledge extracted from Krogh organisms, we consider examples where extrapolation from extreme adaptations could be considered particularly problematic, namely when findings based on extreme adaptations are considered to be relevant and applicable to humans, and hence to have translational power. The following section examines two cases where Krogh organisms are argued to be useful for biomedical research, not because of similarities to human physiology, but because of important differences. In these contexts, Krogh organisms function as ‘negative models’, which is an important feature that further distinguishes some Krogh organisms from standard model organisms.

## Adaptations and translational power: Krogh organisms as negative models

Adaptations of Krogh organisms are sometimes argued to be particularly relevant for human medicine and hence to have what is now commonly referred to as ‘translational power’ (for an overview on the concept of translation, see the contributions to Wehling 2015). How scientists who use standard experimental organisms (particularly rodents) deal with challenges to the validity of their organismal choices, and particularly how they use other components of the research (such as experimental set-ups) to make their choices more plausible, has been explored elsewhere (e.g., Ankeny et al. 2014; Nelson 2018; on ‘plausibility’ see Ankeny and Leonelli in preparation). Here we focus on a narrower but critical question: how can organisms with distinct or extreme adaptations allow for insights that translate to the context of human physiology? To explore this issue, we analyze two examples with different types of outcomes.

The examples we examine in this section are highlighted as Krogh organisms for translational research because of the *absence* of a human disease or disorder. Some biologists have termed this use of experimental organisms as ‘negative models’ of human physiology (Alstrup and Wang 2016). This terminology underscores the idea that organism choice may not only be based on similarities (or representational matching) but also on differences that allow for the exploration of new possibilities. In this context, Krogh organisms can be framed as interesting negative models if they are characterized by the absence of or resistance to physiological problems that humans experience. Interestingly, experiments on ‘negative models’ often include comparisons to similar experiments on human cells or mice that in these contexts represent ‘positive controls’.

Researchers’ interests in negative models, apart from fascination with extreme capacities, are motivated by the hope that understanding why certain physiological limitations are *not* observed in selected species can offer insights into human physiological problems and potential solutions. Adaptations in these contexts thus become heuristics for mechanisms and therapeutic strategies that may help to prevent or cure human diseases. In the following subsection, we examine two types of organisms recognized for their translational utility due to dissimilar features compared to humans, namely mole rats that are cancer resistant and python snakes that are experts in metabolic reactivation of digestive systems.

### Naked mole rats and cancer research

Cancer research often relies on standardized mice and rats as experimental organisms due to their high incidence of cancer and short life spans. While these features make them well suited for testing of carcinogenic effects of chemicals or responses to cancer treatments,^7^ these organisms may not be the best choices if the aim is to discover anticancer mechanisms. As argued in a recent publication in *Nature*, “these traits indicate that mice and rats have fewer anticancer mechanisms, and novel tumour resistance mechanisms are less likely to be discovered using these models” (Tian et al. 2013). Tian and colleagues emphasize that naked mole rats (*Heterocephalus glaber*) are more promising in this respect because they have unusually slow rates of ageing and are cancer resistant, in comparison with mice, rats and other rodents (Seluanov et al. 2008; see also Edrey et al. 2011). The studies cited below highlight the naked mole rat as a useful model due to these differences and rely on parallel studies of human cells or mice as positive controls.^8^

Since cancer is characterized by uncontrolled cell proliferation, researchers have been particularly interested in investigating whether naked mole rats have special defense mechanisms that limit cell growth. Experimental studies of fibroblast cells from naked mole rats have shown that these cells exhibit early contact inhibition (ECI) at much lower cell densities as compared to mouse or human cells (Seluanov et al. 2009). Contact inhibition is a process where cell growth is inhibited by contact with other cells and is considered as an anti-cancer mechanism that is lost in tumor development. The mechanisms of the signals triggering ECI in naked mole rats have recently been identified.

Tian et al. (2013) observed that fibroblast cells of naked mole rats exhibit decreased activity of enzymes that degrade hyaluronan (HA), in comparison to mice and humans. HA is a long unbranched disaccharide polymer that makes up a key component of the extracellular matrix and contributes to the viscosity of the cell (and tumor) microenvironment. The higher rate of HA-degrading enzymes was surprising, since high levels of HA in humans have been associated with increased density and fluid pressure, leading to cell proliferation, inflammation, and increased resistance to chemotherapy (Rankin and Frankel 2016). Naked mole rats, however, excrete high-molecular-mass hyaluronan (HMM-HA), which is more than five times larger than mouse or human HA. The discovery of HMM-HA therefore provided new insights into the importance of the polymer length for the function of extracellular components and opened new avenues for research on mechanisms for modifying cell signaling pathways.

Studies on naked mole rats have led to suggestions for new therapies that target activation of ECI via a HA-induced receptor (CD44) and a pathway involving conserved tumor suppressor proteins (p16^INK4a^) (Seluanov et al. 2009; Tian et al. 2013). In addition to the tumor suppressors found in mice and humans, naked mole rats express a unique protein isoform with higher capacity to induce cell-cycle arrest, and this protein may suggest another potential path for treatment development (Tian et al. 2015). Studies on naked mole rats have also provided insights into the mechanical properties of variants of hyaluronan that influence the viscoelastic properties of the extracellular matrix. These physical properties have been found to influence tumor development and response to cancer therapies, suggesting that attention to soft matter properties of cancer tissues is important for disease prediction and treatment opportunities (Rankin and Frankel 2016). Although it is too soon to determine the clinical utility of suggested treatment opportunities, studies of naked mole rats have provided novel directions for cancer and aging research, some of which may not have been achieved with a different experimental organism (see Sect. 5).

### Snakes as negative models for metabolic problems

Researchers interested in other kinds of human diseases or disorders have similarly argued for the relevance of expanding the scope of experimental organisms beyond traditional model organisms (Alstrup and Wang 2016). Alstrup and Wang argue that it can be of relevance to medicine to understand how giraffes thrive with a blood pressure that would instantly kill humans, or why bears in hibernation do not develop loss of muscular and skeletal functions as do patients in intensive care units. In the following, we examine the choice of a non-standard organism for research on metabolic problems related to the physiological challenge of reactivating the digestive system after long periods without food.

An important physiological adaptation to periods of starvation in many vertebrates is that the intestine shrinks, thus minimizing the energy needed for cell renewal. However, in humans, cell regeneration is often not easily activated. Humans who have experienced long periods of starvation, or who have depended on intravenous nutrient intake during disease treatments, often develop digestive problems because the intestine has been downregulated for too long (Alstrup and Wang 2016). In comparison, most snakes are extremely well adapted to rapidly activate or deactivate their digestive systems. Many species can reactivate their digestive system within a few hours, even after months without eating, and some can digest prey compatible with their own body weight. Ambush-hunting snakes with these capacities have therefore been suggested as a vertebrate model of extreme physiological regulation of organ size and function (Secor and Diamond 1998).

In birds and mammals, the intestine structure is regulated through programmed cell death or apoptosis (deactivation) followed by production of new enterocyte cells in the intestinal crypts (reactivation). The generation of new cells is an energetic bottleneck, and in some cases animals and humans may starve to death even in the presence of food, because the organism does not have sufficient energy to restore the intestine functions required for uptake of nutrients. In contrast, metabolic studies of ball pythons have shown that these organisms are able to regenerate the structure of their intestine without spending much extra energy (Wang et al. 2002; Kjaer 2015). Identification of the mechanisms for the efficient up and down regulation was therefore hypothesized to point to useful pathways to modify during medical treatments, akin to the example of the naked mole rats.

To study the cytological mechanisms that drive the size changes in ball pythons, researchers have employed various imaging techniques, flow cytometry, and immunohistology, using mice as positive controls as in the example with the naked mole rats. Ball pythons are able to shrink their intestines to a third of their size during the inactive state, and to undergo a remarkably fast size increase after feeding (Starck and Beese 2001). In this case, however, the mechanism that was identified turned out to be very different in the Krogh organism compared to the target of translation. Rather than large-scale apoptosis and cell generation, ball pythons can regulate intestine size via a ‘bladder-like’ mechanism that alters the size of enterocyte cells. The snakes are able to inflate or deflate the mucosal epithelium through movement of lipid droplets, resulting in structural changes from folded cell layers to single-layered enterocytes with large surface areas (Starck and Beese 2001; Wang et al. 2002; Kjaer 2015). Thus unlike the regulatory mechanism in mammals, the total number of cells remain almost unchanged. This peculiar mechanism seems to be unique to snakes and can therefore not be generalized to other vertebrates, including humans. For this reason, we consider this example as an interesting test case for discussing criticisms of the Krogh principle.

As briefly discussed earlier in this paper, critics have pointed out that generalizations based on special cases could be problematic because they may neglect the profound diversity in physiological structures and problem solutions found in nature. Krogh organisms cannot be assumed to work as ‘Rosetta Stones’ that are uniquely suited as models for any other species (Gest 1995). Bolker (2009) argues that the Krogh Principle serves as “a warning as well as a positive recommendation: we must be reasonably certain that whatever makes a phenomenon uniquely accessible in a given model is not so peculiar as to prevent generalization”. She clarifies that certain matching criteria between the model and what it represents are essential for inferences to be reliable. While we agree with these points, we have argued that the extent of representational scope cannot possibly be established in advance, but is subject to empirical questioning during the same research process (see also Jørgensen 2001; Sanford et al. 2002). Interpreted as a heuristic, the Krogh principle is a fallible research strategy that points to potentially useful directions but offers no guarantees about potential generalizations.

In this context, resistance to generalization should not be taken as a refutation of the heuristic utility of a Krogh organism. While the discovery of a non-generalizable mechanism for regulation of intestine size is disappointing from the perspective of human physiology, the exploration of the negative model led to the discovery of a new physiological mechanism and insights into a special adaptation in reptiles (Wang et al. 2002). The comparative method thus can also reveal historical relationships between trait developments in different species (see also Wideman and Muñoz-Gómez 2016). Moreover, researchers are still optimistic that other aspects of the extreme metabolic adaptations of snakes can be useful for developing therapies against human diseases (Alstrup and Wang 2016). The Krogh principle should here be understood in the context of what Somero (2000) calls “exploratory physiology”, where variation on common themes (e.g., metabolic problems, and respiration) are investigated to search for solutions that are unknown or not yet well understood.

In general, the utility of the Krogh principle does not in any strong sense depend on a priori assumptions about representational scope. More important for the merits of the principle is whether scientists carefully investigate the scope of application through the comparison of mechanisms in different species. The crucial difference between the examined case with the pythons and the problematic examples highlighted by Krebs and Krebs (1980; see Sect. 1) does not lie in the level of analysis (or in the type of phenomena analyzed). Rather, it lies in the willingness of researchers to take biological diversity seriously. As indicated in the introduction, and as we shall clarify below, the idea that one organism may be a sufficient basis for developing an understanding of physiological problems is in conflict with Krogh’s own work which emphasized the comparative method.

## The comparative method: studying diversity *and* general mechanisms

We have argued that the Krogh principle should be viewed as a heuristic rather than as an empirical claim about optimally suited models that can be utilized as the basis of generalizations. Researchers are often particularly interested in distinct or extreme adaptations because they can ease experimental access to physiological problems or more clearly illustrate relations between environmental constraints and the possible structure and function of physiological traits or mechanisms. While extreme traits clearly constitute non-generalizable special cases, the identified relationships between structure, function, and environment can be useful starting points for exploring physiological possibilities through comparative and contrastive examples. Moreover, they can sometimes suggest possible medical solutions to physiological problems that result in human diseases.

Whether studies of adaptations will result in insights that are possible to generalize or translate cannot be determined in advance, because the representational scope of Krogh organisms is an empirical question that is continuously explored through comparison with other species. Understanding the merits of the comparative approach is therefore important for understanding the merits of the Krogh principle (Robert 2008). A closer examination of the historical context within which Krogh formulated the principle, which was crucially grounded on the comparative approach, can also help to nuance the relation between biological diversity and the search for general mechanisms.

As mentioned in the introduction, Krogh was among the founding fathers of *comparative physiology* which was intended as a bridge between (specialized) human physiology and general physiology. Proponents of the former, including the German pioneer of experimental physiology, Carl Ludwig (1816–1895), emphasized that insights into human physiology could only be based on humans or closely related species (Wang 2011). Accordingly, animal experiments using distantly related or physiologically dissimilar species were considered largely irrelevant to human physiology or medicine. The physiologists Claude Bernard (1813–1878), Peter Ludvig Panum (1820–1885), Christian Bohr (1855–1911), and Krogh (1874–1949) all challenged this view.^9^ The controversy is nicely captured in the following quote by Bernard:

It has been said that experiments performed on a dog or a frog may be conclusive in their applications to dogs and frogs, but never to man, because man has a physiological and pathological nature proper to himself and different. It has been further stated that to be really conclusive for man, experiments would have to be made on man or animals as near to him as possible. (…) How well founded are these opinions? How much importance should we ascribe to the choice of animals in relation to the usefulness of the experiment to physicians? (Bernard 1927, pp. 122–123, quoted in Jørgensen 2001)

The discovery of general mechanisms of homeostasis, respiration, nerve function, and so on from studies of tortoises, frogs, squid, and other organisms documented the relevance for human physiology of experiments on distantly related animals. At the same time, however, Krogh considered general physiology based on a few organisms to be an unrealistic ideal given the diversity of species morphology and adaptations. Krogh disagreed with the assumption of some general physiologists that nature displayed a great generality with only minor modifications (Lindstedt 2014). Acknowledgement of the limitations of generalizing from one species to another was at the heart of Krogh’s own approach, which is also emphasized in his 1929 lecture on the future of physiology:

We will find out before very long the essential mechanisms of mammalian kidney function, but the general problem of excretion can be solved only when excretory organs are studied wherever we find them and in all their essential modifications. (Krogh 1929, p. 202)

The quote shows that Krogh both acknowledged the quest for general or “essential” physiological mechanisms and the importance of biological diversity. His emphasis on the need for systematic comparison of different species underscores his view that the search for generalizations must take the route of studying variation among different organisms (Krogh 1929; see also Jørgensen 2001; Chown and Gaston 2016).

Krogh’s vision of comparative physiology was that it should establish a bridge between human physiology and general physiology by means of the comparative method adopted from zoology. Thus, in Krogh’s view, studies of various “simpler” organisms had much to offer human physiology and medicine. Yet, he considered statements about generality from such organisms dependent on evidence from a broad spectrum of types (Logan 2002). Although Krogh received the Nobel prize for his discovery of a general respiratory mechanism via experiments on frogs and tortoises, he emphasized that essential physiological mechanisms could only be made after careful studies of “the vital functions in all their aspects throughout the myriad of organisms” (Krogh 1929).

Krogh’s comparative approach to the problem of chloride secretion in osmoregulation can serve as an illustration of this strategy. Krogh was interested in the questions of whether and how (freshwater) organisms can take up chloride in very dilute solutions (Schmidt-Nielsen 1995; Chapter 15). Together with his collaborators, Krogh had studied chloride uptake in frogs, crabs, and eels, but the experiments gave inconclusive results. Krogh therefore searched in the published literature for studies on other organisms and discovered that Henrik Lundegårdh, a Swedish botanist, had published results indicating that plant roots were able to absorb and concentrate salts. At the same time, experiments by Henri Koch, a young researcher from Belgium, pointed to a similar mechanism in certain cells in crab gills and insect anal papillae. Krogh invited Koch to visit his laboratory and together they were able to give conclusive experimental evidence of such a mechanism in insect larvae (Koch and Krogh 1936). The year after, Krogh published a note in *Nature* (1937) where he argued for the similarity of a mechanism for chloride uptake and concentration based on a review of multiple studies across the animal kingdom. Together with the English physician V. B. Wigglesworth, Krogh improved the experimental techniques to study ion transport across cells and cell membranes. Krogh showed that the mechanism for absorbing and concentrating chloride only operated in animals under salt depletion, which not only explained some of the inconclusive results but also showed that the mechanism in animals is regulated.^10^

Most of Krogh’s writings combined a review of other studies on various species with presentation of his own finished and unfinished experimental studies. In the discussions, he compared mechanisms in different species and reflected on the degree to which mechanisms were general or specialized adaptations (see e.g., Krogh 1916, 1941, 1946). This underscores that the interest in diversity needs not be in tension with the quest for general principles but can even be a route to these.

The comparative method has recently gained philosophical attention through Wouters (2007) account of so-called design explanations. Discussing the implications of this type of scientific explanations is beyond the scope of this paper, but we shall highlight an important point from Wouters’ paper concerning the significance of the comparative approach. To clarify how the study of diversity can lead to insights into general dependence relations between structures and functions, Wouters (2007) considers the constraints on respiratory systems for different organisms. As mentioned, Krogh showed that gas exchange during respiration does not rely on active transport but happens via diffusion. Accordingly, all respiratory “designs” must obey the constraints given by Fick’s law of gas diffusion which states that the diffusion rate is proportional to the surface area and the concentration gradient. Given this background, important questions for the (comparative) physiologist is to understand how it is possible for different types of organisms to survive in different types of environments. Depending on the size of the animal and environmental constraints, certain types of physiological solutions are possible (e.g., respiration via lungs, gills, or open respiratory systems), and these types display further variation according to environmental demands. Consequently, Wouters argues that the comparative method not only provides insights into specific adaptations, but also into more generic types of solutions or functional dependence relations.^11^

In line with this use of the comparative method, one strand of research inspired by Krogh’s work emphasizes unity in diversity (Somero 2000; Dobson 2014; Abzhanov et al. 2008). Examples of unifying principles of biological design discovered through comparative studies are homeostasis or allometric scaling relations. Krogh’s former student and son-in-law, Knut Schmidt-Nielsen (1915–2007), stressed in multiple publications the need to explain why power law scaling relations of metabolic rates can be identified across different species. When the body mass is plotted on a logarithmic scale against basal metabolic rate, the points of birds and mammals fall on a single straight line (Schmidt-Nielsen 1984: 57). Why this is the case, to which extent such general relations hold, and whether such relations should be described via systemic, physical, or cell-based approaches are ongoing issues of controversy in biology (Wouters 2007; Glazier 2015; West 2017). Comparative physiology thus explores the productive tension between experimental studies of diversity at different levels of organization and theoretical considerations about more general constraints on biological diversity.

The comparative method also involves careful examination of specific physiological and environmental constraints, which may facilitate an understanding of *why* general principles apply in different contexts where we would not expect physiological similarities. For example, it was recently discovered that the signal design of echolocation in bats and whales are strikingly similar, despite the huge size differences of these animals. This finding was surprising because relationships between directionality and frequency of clicks depends on the size of the sound emitter. Accordingly, the size differences of bats and whales should be reflected in differences in sound frequencies. However, comparative studies have shown that both types of organisms have arrived at a similar signal design because they must balance opposing physical constraints presented also by their respective terrestrial and aquatic environments (Madsen and Surlykke 2013). Without the comparative method, the applicability of the general model for signal design would be unexplained. Thus the comparative analysis explains *why* the principles are the same in the two cases by appealing to functional constraints within which the physiological structures and behaviors must function.

## The biomedical relevance of extreme organisms

A final question to consider is whether there is a risk in assuming that the quest for general mechanisms is compatible with the fascination with ‘extreme’ organisms. How is it possible to justify that organisms with distinct or extreme adaptations are useful for general physiology or biomedicine? As argued in Sect. 2, the focus on extreme organisms is premised on the idea that these provide clear illustrations of relationships between structural, functional, and environmental constraints. As stressed by contemporary comparative physiologists: “In these [studies of physiological adaptations] we seek to understand the general through the extreme, because animals with specific adaptations make it easier to understand the mechanisms that make possible survival under extreme conditions” (Alstrup and Wang 2016: 2, our translation). Studying the scope of variation in physiological mechanisms or structures such as kidney morphology under different environmental conditions and in different species can thus facilitate insights into the “constraints on being alive” (Wouters 2007, p. 66) and help us to understand why animals in different environments may rely on different functional designs.

Recently, appeals to the Krogh principle have pointed to the advantages offered by studies of organisms that are not traditional model organisms, in arguing for the importance of maintaining diversity of organisms in experimental biology and medicine (Burggren 1999; Logan 2002; Beery and Kaufer 2015). Some emphasize that Krogh organisms can compensate for or counteract the biases present in the narrow focus on traditional model organisms. Beery and Kaufer (2015: 117) argue that “modern biological research is strongly biased towards rat and mice; in 2009 rats and mice made up approximately 90% of mammalian research subjects in physiology, up from 18% at the time Krogh’s principle was articulated”. Similarly, Love (2010) emphasizes that Krogh organisms can serve as a useful compensatory tactic in developmental biology because they are likely to expose blind spots in the idealization strategies associated with standard models of developmental staging. Traditionally, organisms are selected for developmental research because they exhibit comprehensive and generalizable ‘normal stages’ (Lowe 2015). To balance out use of experimental organisms exhibiting minimal phenotypic variation and plasticity, a comparative approach involving contrasting cases can provide a useful complementary perspective that exposes the scope of variation in other organisms.

Considerations of potential blind spots may be particularly important in contexts where research funding is directed towards use traditional model organisms in order to maximize the return on prior investments in genome sequencing or databases (Bolker 2009; Bolker and Brauckmann 2015). This focus is not necessary biased, but some have appealed to the Krogh principle when highlighting that an overly narrow focus may lead to missed opportunities:

Today, the main part of biomedical research is limited to very few species (mice and rats alone make up about 85 percent of all vertebrates in animal experiments). With about 60.000 vertebrates (besides 30-50 million invertebrates), science should maybe reconsider if there are other and better experimental organisms for our attempt to understand how the body functions and what goes wrong during disease development. (Alstrup and Wang 2016: 6, our translation)

Thus alternative models can provide insights into mechanisms that would not have been discovered using traditional model organisms. Burggren (1999) provides an excellent example of how the exploration of alternative experimental organisms in developmental biology led to the discovery of animals with transparent eggs or embryos that allowed for important experimental observations (see also Burggren and Warburton 2007). Similarly, a commentary on the case presented in Sect. 3, on cancer research on mole rats, emphasizes that some opportunities are only discovered when studying non-model organisms:

Would they have found any of this out without studying the naked mole rat? Absolutely not. Once again, the wisdom of the Krogh principle shines through: Studying similarities and differences between ourselves and other animals can teach us a lot about the function of our own bodies in health (physiology) as well as in disease (pathophysiology). (Lee 2013)

The modifications that make naked mole rats resistant to cancer may be unique to this species and may not have been discovered without these studies. Nevertheless, the molecular pathways and modifications that have been discovered in them may be sufficiently similar to human physiology to suggest new treatment opportunities. Even in cases where translation is not possible, such as in the case with digestive mechanisms in pythons (Sect. 3), non-model organisms may give insights that are physiologically interesting in their own right. Thus, Krogh organisms are often chosen simultaneously for their translational potential and for the value of comparative physiology “for its own sake” in providing a deeper understanding of the scope of diversity within physical and physiological constraints (see also Schmidt-Nielsen 1995; Chapter 15). In his seminal 1929-lecture, Krogh explicitly highlighted that his proposal of comparative physiology at the same time was more relevant for medicine and more independent from it:

Physiology as a science has taken its origin from the necessities of practical medicine, and even now the large majority of workers in physiology have had the benefit of a medical education and hold their appointments in medical schools. Nevertheless we all hold physiology to be an independent science, and most of the work done in physiological laboratories has no direct relation to medicine. The line of development which I think should be followed is to establish in one direction a branch of physiology which is much more intimately in contact with practical medicine and in the other direction a branch which is much more independent. (Krogh 1929, p. 202)

In other words, Krogh proposed a field of comparative physiology that on one hand was more motivated by physiological problems of relevance to medicine. But he also highlighted that by studying organismal diversity that is *dissimilar* to human physiology it is often possible to discover “adaptations of exquisite beauty and the most surprising character” (Krogh 1929, p. 203). Examples of such diverse and surprising adaptations are provided in his three monographs, entitled *The respiratory exchange of animals and man* (1916), *Osmotic Regulation in Aquatic Animals* (1939) and *The Comparative Physiology of Respiratory Mechanisms* (1941). The span of examples allows for the books to contribute simultaneously to human and animal physiology.

In summary, the Krogh principle does not entail a belief that a single organism is suitable for providing all answers to a given physiological problem. An examination of the historical background and current appeals to the principle in comparative physiology reveals that the principle is tied to an investigation of zoological diversity, explored through the comparative method. Moreover, we have highlighted common characteristics that distinguish Krogh organisms from model organisms. Traditional model organisms have been characterized by their wide representational target and scope, and the use of standardization procedures and data infrastructures to ground the validity of the model as a reference point for cross-species inference. Krogh organisms can instead have a highly variable representational scope, determined by the context of use, and are often characterized by distinct or extreme adaptations that facilitate experimental access and theoretical understanding of specific physiological problems. In the context of biomedical research, Krogh organisms can help balance the focus on standardized organisms and offer insights to disease defensive mechanisms that are absent in humans and closely related organisms.

## Conclusion

Through reference to examples of how the Krogh principle has been used in biological practice, we have argued that so-called Krogh organisms as used in experimental biology have virtues that are complementary to traditional model organisms. In contrast to model organisms, they serve as experimental access points for the identification and exploration of specific mechanisms or physiological problems that are particularly pertinent or accessible in these organisms. By selecting experimental animals where a trait is more accessible or apparent, causal relations between the trait and the environment are easier to pick out and hence make the identification of solutions to physiological problems clearer.

In response to criticisms of the Krogh principle and concerns about representational limitations of Krogh organisms, we have focused on clarifying why organisms with distinct or extreme adaptations are sometimes considered to be experimentally useful for purposes of exploring general mechanisms or for translational biomedical research. Aside from offering experimental access to identify solutions to physiological problems where these are most distinctively displayed, a focus on adaptations, such as the kidney of desert rats, can provide insights into general relations between physiological structures and functions and environmental constraints that organisms must obey to stay alive. Importantly, however, we have argued that assumptions around the representational scope of Krogh organisms need to be empirically investigated and critically tested through the comparative method. That is, the generality of the discovered mechanism must be explored through comparison with organisms that have different morphological characteristics or are exposed to different environmental constraints. We believe that this interpretation is in line with Krogh’s intentions when he proposed his seminal principle.

When the Krogh principle is criticized for leading to unwarranted generalizations from optimal models, it is thus important to consider whether the principle has become detached from the comparative method. Lack of attention to this part of the heuristic may also lead to the (mistaken) view that there is a dichotomy between the study of diversity and the quest for generalizations. An important lesson from Krogh’s work, as well as from contemporary work in comparative physiology, is that insights leading to generalizations often occur via studies of a diverse range of solutions to address common physiological problems. Comparative physiology explores the productive tension between generalizations stemming from constraints on possible designs of organisms and the great diversity of organisms.

## Footnotes

1 In 1910, Krogh was granted funding by the Danish Parliament to open his own research institute, the University Zoophysiological Laboratory, which became an important venue for the development of comparative physiology. The institute was supported by the Insulin Foundation until a few years after Krogh’s death (Schmidt-Nielsen 1995).

2 We here use the term ‘mechanism’ in a wide sense, following how biologists often use the term as a short-hand for processes or characteristics (physiological or molecular) that are responsible for life-sustaining functions (see also Wimsatt 2007: 173). Thus, our use of the term does not presuppose a specific philosophical account of mechanistic explanations or refer to any specific level of analysis.

3 This claim is based on our examination of over 250 papers that refer to the Krogh principle. We have labelled these according to the justifications given for organism choice (such as extreme or distinct adaptations, experimental convenience, evolutionary reasons, generalizability, translational power, ethical reasons, and so on). What we highlight here are most commonly provided scientific justifications which we contend are in need of philosophical analysis. In a separate paper, we pay more attention to the diversity of uses of the principle.

4 For a discussion on the historical development of the experimental studies and the debate on oxygen secretion, see (Schmidt-Nielsen 1995, Chapters 9 and 11; Wang 2011).

5 Some authors appeal to the Krogh principle when justifying organism choice that is suitable for addressing evolutionary questions, and physiological and evolutionary studies often go hand in hand. For instance, fish have been used as experimental organisms to understand the evolution of oxygen chemoreception in other vertebrates, including mammals, because these historically and in current environments experience extremes in low oxygen availability (Burleson 2009; see also Logan 2002; Love 2010). However, because many uses of the Krogh principle focus primarily on proximate causes, we focus in this paper on the use of experimental organisms for addressing physiological questions, some of which are guided by evolutionary considerations.

6 For a general discussion of heuristics in biological research, see Wimsatt (2007).

7 Recently, the utility of experimental animals for toxicological studies has been debated due to important dissimilarities, and hence studies on human cells or computer simulations have been argued to be more promising (May 2009; Shanks et al. 2009; van der Worp et al. 2010). We shall not engage in these debates here as we are primarily interested in exploring whether dissimilar organisms also can be useful for biomedical research, and if so, why.

8 Importantly, whether something is framed as a standard or model organism or a Krogh organism depends on the specific problem focus. Alstrup and Wang (2016) for instance highlight that mice can serve as negative models for research on Alzheimer’s disease because these do not develop the disease unless specific human genes are incorporated into their genome.

9 Jørgensen (2001) points out that the similarity in viewpoints may have been influenced by mentoring relations between the four. Krogh’s mentor, Bohr, worked as lab assistant under Panum, the first Danish professor in physiology, who also stressed the importance of comparative anatomy (see also Schmidt-Nielsen 1995, Chapter 5). Panum’s views have likely been influenced by interactions with Bernard as he had spent a year as his laboratory in France.

10 For a discussion of this work in comparison to recent insights into active ion transport, see (Schmidt-Nielsen 1995; Chapter 15, particularly note 25).

11 For a discussion of how design explanations and other generic explanations relate to mechanistic explanations, see (Braillard 2010; Green and Jones 2016).
